# Small power and lighting load time series data for 27 departments across 8 UK hospitals

**DOI:** 10.1016/j.dib.2016.03.076

**Published:** 2016-03-30

**Authors:** Paula Morgenstern, Maria Li, Rokia Raslan, Paul Ruyssevelt, Andrew Wright

**Affiliations:** a UCL Energy Institute, University College London, 14 Upper Woburn Place, London WC1H 0NN, United Kingdom; b Troup Bywaters and Anders, 183 Eversholt Street, London NW1 1BU, United Kingdom; c UCL Institute for Environmental Design and Engineering, University College London, 14 Upper Woburn Place, London WC1H 0NN, United Kingdom; d Institute of Energy and Sustainable Development, De Montfort University, The Gateway, Leicester LE1 9BH, United Kingdom

**Keywords:** Energy, Hospitals, Laboratories, Operating theatres, Energy management, Lighting

## Abstract

The electricity consumption of 27 departments was measured across 8 medium to large General Acute hospitals in England (largely by the authors, some data was donated and authorised for publication by the respective hospitals). The departments fall into 6 different categories which have been selected due to their prevalence in General Acute Hospitals (wards), their high energy intensities (theatres, laboratories, imaging and radiotherapy) or their distinct operating hours (day clinics). This data article provides floor areas and the time series of departmental power loads, mostly encompassing lighting and small power (but excluding central electricity use for ventilation, pumping and medical gas services). Comparative interpretations of the data are published in doi: 10.1016/j.enbuild.2016.02.052 [1].

**Specifications Table**

**Table:** 

Subject area	*Built environment*
More specific subject area	*Energy demand in hospitals*
Type of data	*Excel file*
How data was acquired	*Electrical measurements were carried out using various types of measurement equipment:*
	• *CurrentCost EnviR Energy Monitor*
	• *HOBO UX120-006Ms with CTV-C (10–100 A)*
	• *Dent Data Pro*
	• *Dent Elite Pro (0–6000 A, 80–600 V)*
	• *Profile*
	*For some hospitals, automatic meter readings were available on the energy management system.*
Data format	*Raw*
Experimental factors	*Data collection in-situ, no preparation/pre-treatment*
Experimental features	*Interval measurement of currents through current clamps, Estimation of power loads based on measured currents and typical voltages and power factors*
Data source location	*England*
Data accessibility	*Data with this article*

**Value of the data**

• Very little measurement data of hospital energy use are currently available in the public domain. Given this dearth of evidence, the data published here represent a fairly extensive set of measurements.
• The data can be used for researchers investigating hospital energy use to compare and benchmark their measurements as well as for facilities management staff in hospitals.
• The data further allows for a characterisation of energy intensive and less energy intensive departments as well as operational characteristics, potentially providing a guiding framework to innovative carbon mitigation strategies in the future.

## 1 Data

The electricity use of 27 departments was measured across 8 medium to large General Acute hospitals in England between 2009 and early 2015. The majority of the load time series were measured by the authors, some data was donated and authorised for publication by the respective hospitals. Details on the data source for each department are published in [1].

The investigated departments fall into 6 different categories which have been selected for analysis due to their prevalence in General Acute Hospitals (wards), their high energy intensities (theatres, laboratories, imaging and radiotherapy) or their distinct operating hours (day clinics). Function and operational characteristics of the departments were confirmed through site audits, while floor area values were established from floor plans.

## 2 Experimental design, materials and methods

For some of the departments, automatic meter readings were available on the trust׳s energy management system. For all others, electrical measurements have been carried out at the distribution boards serving the respective departments using various types of equipment (see [1] for details of measurement equipment). With the devices that only measure current, it is assumed the voltage is 240 V and the power factor is 0.9 based on typical known on-site voltages in urban areas and typical reactive components in hospitals [2]. Many hospital departments were served by two distribution boards, traditionally labelled essential (E) and non-essential (NE). Historically, only the essential board was backed up by emergency generators but this separation has become obsolete in many hospitals with the expansion of emergency generator power to cover (almost) all loads. Total departmental SP&L load was the sum of all distribution boards.

Loads were monitored for a minimum of 7 days to capture day of week (particularly weekend) influence on electricity use. If possible, monitoring was continued for up to 4 weeks to increase the reliability of the measurement. It is focussed on local electricity use for power (including fan coil units if present) and lighting but excluding central services, i.e. ventilation and cooling.

There were a number of practical limitations to obtaining the presented measurement data to be taken into account when using the provided data. They are outlined in detail in [1]. Importantly, there is a systematic error from frequently only measuring currents instead of all power characteristics while the comprehensive use of energy analysers was not possible due to funding constraints.
